# Papaverine loaded injectable and thermosensitive hydrogel system for improving survival of rat dorsal skin flaps

**DOI:** 10.1007/s10856-023-06732-4

**Published:** 2023-05-20

**Authors:** Md Sowaib Ibne Mahbub, Yeong jin Kim, Hwanjun Choi, Byong-Taek Lee

**Affiliations:** 1 Department of Regenerative Medicine, College of Medicine, Soonchunhyang University, Cheonan, South Korea; 2 Department of Plastic & Reconstructive Surgery, Soonchunhyang University Hospital, Cheonan, South Korea; 3 Institute of Tissue Regeneration, College of Medicine, Soonchunhyang University, Cheonan, South Korea

## Abstract

Vasospasm during reconstructive microsurgery is a common, uncertain, and devastating phenomena concerning flap survival. Topical vasodilators as antispasmodic agents are widely used to reduce vasospasm and enhance microvascular anastomosis in reconstructive microsurgery. In this study, thermo-responsive hydrogel (CNH) was fabricated by grafting chitosan (CS) and hyaluronic acid (HA) to poly(N-isopropylacrylamide) (PNIPAM). Papaverine, an anti-spasmodic agent, was then loaded to evaluate its effect on rat skin flap survival. Post-operative flap survival area and water content of rat dorsal skin flap were measured at 7 days after intradermal application of control hydrogel (CNHP0.0) and papaverine loaded hydrogel (CNHP0.4). Tissue malondialdehyde (MDA) content and superoxide dismutase (SOD) activity was measured using enzyme linked immunosorbent assay (ELISA) to determine oxidative stress in flaps. Hematoxylin and eosin (H&E) staining and immunohistochemistry (IHC) were performed to evaluate flap angiogenesis and inflammatory markers. Results showed that CNHP0.4 hydrogel could reduce tissue edema (35.63 ± 4.01%), improve flap survival area (76.30 ± 5.39%), increase SOD activity and decrease MDA content. Consequently, it also increased mean vessel density, upregulated expression of CD34 and VEGF, decreased macrophage infiltration, and reduced CD68 and CCR7 expression based on IHC staining. Overall, these results indicate that CNHP0.4 hydrogel can enhance angiogenesis with anti-oxidative and anti-inflammatory effects and promote skin flap survival by preventing vascular spasm.

**Graphical Abstract:**

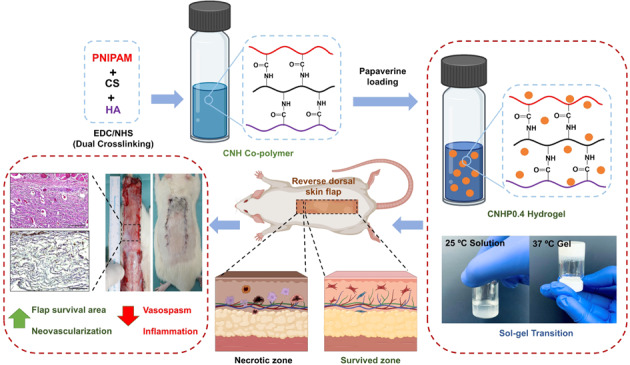

## Highlights

- Thermo-responsive hydrogel (CNH) was fabricated by grafting chitosan (CS) and hyaluronic acid (HA) to poly(N-isopropylacrylamide) (PNIPAM).
- Papaverine, an anti-spasmodic agent, was loaded to evaluate its effect on McFarlane’s rat dorsal skin flap model.
- The hydrogel did not elicit acute toxicity in-vitro and in-vivo.
- Slow release of the drug from the hydrogel in the flap injury site can reduce vasospasm and subsequently promote skin flap survival.
- The hydrogel can significantly increase neo-vascularization, decrease oxidative stressed, and decrease inflammatory reactions.

## Introduction

Skin tissue defects caused by trauma, diabetes, cancer, and congenital deformities are common cases in plastic and reconstructive surgeries. Random skin flaps are used for transplantation in this kind of skin defects for reconstruction [1, 2]. However, flap necrosis on distal end is a frequent complication after surgeries. Oxidative stress, insufficient blood supply and inflammatory reactions have been reported to contribute to flap necrosis [3–5]. Therefore, inhibiting oxidative stress, promoting angiogenesis, and reducing inflammatory cytokines are leading approaches to increase the survival rate of random skin flaps.

Papaverine is a popular choice of antispasmodic drug used topically or directly to blood vessels to treat vasospasm. Papaverine acts by inhibiting cyclic nucleotide phosphodiesterase (PDE), causing an increase in cyclic guanosine monophosphate (cGMP) that can reduce contraction of vascular smooth muscle [6]. In reconstructive microsurgery, it is used as a smooth muscle relaxant to reduce vasospasm and microvascular thrombosis [7] resulting angiogenesis. However, prolonged administration is advised to maintain long-term efficacy because papaverine has a limited half-life of 1.5 to 2 hours [8]. Therefore, efforts to create control-release drug platforms that guarantee its slow release are encouraged in order to address this problem.

Recently, interest in intelligent biopolymer systems with in situ gel-forming capability to develop medical implants and drug delivery systems has increased [9, 10]. Poly(N-isopropylacrylamide) (PNIPAM) is a well-known synthetic thermo-responsive polymer with reversible sol-to-gel phase transition activity in water below its critical temperature (LCST) [11]. In order to bring the gelling temperature closer to physiological level and make the hydrogel more biocompatible, it can be combined with chitosan, collagen, hyaluronic acid, gelatin, and other natural polymers [12, 13]. PNIPAM-based copolymer hydrogels are injectable, allowing for administration by a syringe into a surgical cavity which is simple, less expensive and time saving procedure [14]. Chitosan (CS) is widely used in the field of tissue engineering because of its remarkable bioactivity, non-toxicity, antioxidation, mucoadhesive property, biocompatibility, and simplicity of modification via hydroxyl and amine groups [15–17]. Chitosan has received a lot of attention from scientists working in the field of tissue regeneration as a potential biomolecule [18]. Hyaluronic acid (HA), a natural polymer and major component in extracellular matrix known for its water absorption and retention abilities, can exert lubrication at its application site [19]. Utilizing sol-gel phase transition characteristics of PNIPAM, we grafted CS and HA to make CNH co-polymer and loaded papaverine. The copolymer hydrogel could act as drug carrier system. It can be injected easily into an irregularly damaged tissue cavity. Upon gelation of the injected hydrogel at physiological temperature, papaverine could release slowly and exert its effect on injury site.

Therefore, after fabricating the CNH co-polymer, evidence of modification and phase transition behavior was verified. Papaverine was loaded with CNH to form a thermosensitive CNHP0.4 hydrogel. It was then subjected to in-vitro cytotoxicity evaluation and finally applied intradermally in random skin flaps. We investigated the antispasmodic effect of papaverine loaded hydrogel on flap survival rate, tissue edema, neo-vascularization, inflammatory reactions, and oxidative stress via histological, immunohistochemistry (IHC), and enzyme linked immunosorbent assay (ELISA) analyses, respectively.

## Materials and methods

### Materials

Carboxylic acid terminated poly(N-isopropylacrylamide) (PNIPAM, average Mn 10,000), medium molecular weight chitosan (Mw- 190,000–310,000 Da), sodium salt of hyaluronic acid obtained from *Streptococcus equi*, 2-morpholinoethane sulfonic acid (MES), and papaverine hydrochloride (purity ≥98%) were purchased from Sigma-Aldrich Co, USA. 1-Ethyl-3-(3-dimethylaminopropyl) carbodiimide (EDC) and N-hydroxysuccinimide (NHS, assay ≥98%)) were obtained from Sigma-Aldrich Co., USA. L929 fibroblast cell line was purchased from the American Type Culture Collection (ATCC) and used for in vitro studies. Eagle’s minimum Essential Medium (CORNING), fetal bovine serum (FBS; Thermo Fisher Scientific, USA), penicillin (100 U/ml) and streptomycin (100 U/ml) (PS; Thermo Fisher Scientific, USA). Gibco™ trypsin-EDTA (Thermo Fisher Scientific, USA), Gibco™3-[4,5-dimethylthiazol-2-yl]-2,5 diphenyltetrazolium bromide (MTT; Thermo Fisher Scientific, USA), Dimethyl sulfoxide 99.5% (DMSO; Daejung Chemicals & Metals CO., Korea), Bovine Serum Albumin (BSA; Sigma-Aldrich, USA), ethyl alcohol anhydrous 99.5% (Daejung Chemicals & Metals CO., Korea), and phosphate buffer saline tablets (PBS; Sigma, USA) were used in in vitro studies. For an in vivo study, 12-week-old male Sprague-Dawley rats (*Rattus norvegicus)* (250 to 300 g each) were obtained from DBL (Chungcheongbuk, Korea). All animal experiments and protocols were reviewed and approved by the Animal Ethical Committee of Soonchunhyang University, South Korea (approval number-SCH-0062).

### Synthesis of CNH co-polymer

For the synthesis of CNH co-polymer, carboxylic acid terminated poly(N-isopropylacrylamide) (PNIPAM) and chitosan were first dissolved in 50 ml of 0.1 M MES buffer (pH 5.0) according to concentrations mentioned in Table S1 along with EDC and NHS. The reaction mixture was kept under intense stirring at room temperature for 12 hours and purified by thermo-precipitation for 30 minutes at 50 °C in the presence of 0.6 M NaCl followed by centrifugation at 12,000 rpm for 15 minutes. The precipitate was again dissolved in MES buffer and repeated three times for removal of unreacted CS. This grafted polymer and 2% w/v HA were dissolved in 50 ml of 0.1 M MES buffer (pH 5.0) in the presence of EDC and NHS. The same procedure described above was repeated to obtain CNH co-polymer. Finally, it was dialyzed against distilled water for 4 days at 4 °C and lyophilized to yield CNH co-polymer.

### Fourier transform infrared spectroscopy (FTIR),

FTIR spectra of raw polymers (PNIPAM, chitosan and hyaluronic acid) and synthesized co-polymer (CNH) were evaluated with an FTIR (Thermo Scientific Nicolet iS10) spectrometer. Spectra of Chitosan, PNIPAM, hyaluronic acid, and CNH were recorded in the wavenumber range of 4000 cm^−1^ to 650 cm^−1^.

### Preparation of CNHP0.0 hydrogel and papaverine loaded CNHP0.4 hydrogel

Initially, different concentrations (3%, 4%, and 5%) of CNH co-polymer were dissolved in distilled water to yield hydrogels and evaluated with gelation time. The 5% co-polymer solution was considered as the optimal concentration for this study and named as CNHP0.0 hydrogel. Papaverine hydrochloride was then loaded with optimized 5% w/v CNH-co-polymer solution at 0.4 mg/ml concentration by simple mixing at 4 °C for 4 hours that was named as CNHP0.4 hydrogel and stored for further use. The concentration of papaverine was calculated by considering body mass index (BMI) of rat [20] in comparison with human. In human skin flap surgery, 30 mg/ml single shot papaverine is regularly used [21].

### Phase transition behaviour of developed hydrogel

The sol-gel phase transition behavior of CNH-hydrogel was evaluated by a vial inversion method. In brief, 1 ml of CNH solution was taken in a tightly caped glass vial and kept at 4 °C for 10 minutes. Gel formation time was then recorded using a stopwatch after heated in a 37 °C temperature-controlled system.

### Viscosity measurement

The viscosity of CNH hydrogel was measured at room temperature (25 °C) and body temperature (37 °C) with a Brookfield viscometer (DV2T, USA) at different shear rates (0.068, 0.136, 0.204, 0.272, 0.34, 0.51, and 0.68 sec^−1^).

### Water content and volume shrinkage

Comparative water contents of raw polymer, PNIPAM, and synthesized co-polymer CNH were determined by placing 1 ml of 5% (w/v) solution in a 37 °C incubator for 1 hour followed by addition of another 1 ml extra water to ensure wettability of hydrogel. After 24 hours, the wet hydrogel was weighted and water content was measured with the following equation:

$${{{\mathrm{Water}}}}\,{{{\mathrm{content}}}} = \frac{{{{{\mathrm{W}}}}_{{{\mathrm{h}}}} - {{{\mathrm{W}}}}_p}}{{{{{\mathrm{W}}}}_p}}$$

where W_h_ was the weight of hydrogel at 37 °C and W_p_ was the dry weight of the polymer.

The percent volume shrinkage of each hydrogel was determined by the loss of volume after phase transition using the following equation:

$$\begin{array}{*{20}{c}} {{{{\mathrm{Volume}}}}\,{{{\mathrm{shrinkage}}}}\left( {{{\mathrm{\% }}}} \right)} & = & { - \left( {1 - \frac{{V_{{{\mathrm{I}}}} - V_{H2O}}}{{V_{{{\mathrm{I}}}}}}} \right) \times 100{{{\mathrm{\% }}}}} \\ {} & = & { - \left( {1 - \frac{{V_W}}{{V_{{{\mathrm{I}}}}}}} \right) \times 100{{{\mathrm{\% }}}} = - \frac{{V_{H2O}}}{{V_{{{\mathrm{I}}}}}} \times 100{{{\mathrm{\% }}}}} \end{array}$$

where V_I_ was the initial volume of polymer solutions at room temperature (25 °C), V_H20_ was the squeezed water volume after gel formation, and V_W_ was the water volume in hydrogel at 37 °C.

### Determination of lower critical solution temperature (LCST)

LCSTs of PNIPAM and CNH were determined using a temperature-controlled UV/VIS optical spectrophotometer. Both polymers were prepared in distilled water to have 5% w/v solutions. The absorbance was then taken every two minutes at wavelength of 470 nm from 20 °C to 40 °C after equilibrated at each test temperature. From the temperature-absorbance curve, the temperature at which absorbance was half of the maximum value was determined as the LCST of each polymer.

### In-vitro papaverine release from hydrogel

Papaverine release from hydrogel was evaluated using the dialysis tube diffusion method. A 12,000–14,000 molecular weight cut-off dialysis tube purchased from SERVA Electrophoresis GmbH, Germany was used for this experiment. The tube was hydrated with PBS (receptor medium, pH 5.5) before placing the drug loaded hydrogel in it. Freshly prepared CNHP 0.4 hydrogel (2 ml) was placed in the dialysis tube. The tube was then placed in a beaker containing PBS (30 ml receptor medium, pH 5.5). The diffusion system was maintained at 37 °C temperature with continuous stirring with a magnetic stirrer. After pre-defined time intervals, 2 ml of defused receptor medium was withdrawn and refilled with equal amount of PBS (receptor medium, pH 5.5). All withdrawn aliquots were measured at 254 nm with a UV spectrophotometer.

### In-vitro cytotoxicity evaluation

MTT assay was carried out to evaluate the cytotoxicity of papaverine loaded (CNHP0.4) hydrogel or hydrogel not loaded with papaverine (CNHP0.0) to L929 cells cultured in ATCC recommended media (EMEM, 10% horse serum, 100 U/ml 1% penicillin, and 100 U/ml streptomycin). Hydrogels were sterilized in dry state under UV exposure for 3 hours. Prepared hydrogels were then kept in EMEM culture media at 37 °C for 24 hours to obtain hydrogel extracts for indirect assay according to ISO 10993-5. 24-well cell culture plates were seeded with 1 ml cell suspension (2 × 10^4^ cells/well) and allowed to attach for 24 hours, culture media were replaced with extraction media and cultured at 37 °C with 5% CO_2_ for 1, 3, and 7 days. At these time intervals, 5 mg/ml 100 µl MTT solution was added to each well and incubated at 37 °C for 4 hours to form formazan. Without disturbing the layer of formazan, MTT solution was aspirated and DMSO was introduced to dissolve the formazan by incubating at 37 °C with agitation for another 30 minutes. The absorbance was measured at 570 nm with a multiplate reader (PerkinElmer, Victor^TM^X3).

Another set of culture plates under the same culture conditions were used to evaluate cell proliferation. At pre-determined time intervals, cells were fixed with 4% paraformaldehyde for 15 minutes. Fluorescein isothiocyanate conjugated Phalloidin (FITC, Sigma-Aldrich) and Hoechest 33342 (Invitrogen, USA) were used to stain actin and nuclei of cells followed by permeabilization and blocking with Triton-X100 (Bio-Rad, USA) and bovine serum albumin, respectively, followed by visualization under a fluorescent microscope (Nikon, ECLIPSE-Ti2, Tokyo, Japan) coupled with an NIS-Elements AR 5.20 Viewer software.

### In-vivo study with rat dorsal skin flap model

Twenty 12-week-old male Sprague-Dawley rats (*Rattus norvegicus)* were divided into two groups (with and without papaverine loaded hydrogels) in this experiment. Rats were maintained in individual cages at air-conditioned temperature (25 °C) with sufficient food and water. They were anesthetized using isoflurane (Terrel, USA) with oxygen and N_2_O gas. For maintaining the anesthesia, 3% isoflurane was continuously administered. In this study, Reverse type McFarlane’s rat dorsal skin flap model was used to investigate effects of different factors on skin flap survivability. The hair on the back of rats were carefully shaved and outlined 9 cm × 3 cm as shown in Fig. 4a. Flaps were elevated up to hip joints as the indicator of baseline of flaps and immediately sutured using 4-0 nylon sutures (AILEE Co., Korea) after intradermal application of only hydrogel (CNHP0.0) or hydrogel with papaverine (CNHP0.4) to two groups of rats. Total flap was divided into three equal parts and named as Zone-I, Zone-II, and Zone-III. Tramadol and enrofloxacin were given intramuscularly to relief pain and prevent infection, respectively, after surgery. Animals were kept in individual cages with a neck collar to prevent self-cannibalization. All rats were euthanized by isoflurane overdose at 7 days after operation.

### Flap survival area measurement

At postoperative day 7, photographs were taken with a Canon EOS 90D (Tokyo, Japan) camera from a standard distance to assess flap survival and necrosis area. Dark and remaining zones of flaps were identified as necrotic and viable areas, respectively. They were measured with a Fiji-ImageJ software. Viable area of the flap was calculated by subtracting necrotic area from total area of the flap. It was expressed as percentage of flap survival area.

### Tissue water content measurement

Tissue water contents were measured from the extracted flaps by dehydrating the pre-weighted flaps at 50 °C in an autoclave. Samples were weighted regularly until there was no change in weight for two days. Tissue water content was also expressed in percentage.

### Histological examination

Dorsal skin flaps were collected by sacrificing rats at 7 days after operation. Skin samples of 1 cm × 1 cm from zone-II were biopsied and fixed in 4% paraformaldehyde. All samples were washed, dehydrated, embedded in paraffin blocks, and section into 5 µm-thick slices with a microtome (Thermo Scientific, USA). They were finally stained with hematoxylin and eosin (H&E) (Abcam) following the manufacturer’s instructions. Stained tissue slides were observed under a light microscope (Olympus, BX53). Images were captured with a camera (Olympus DP72) using Cellsens software. Mean vessel density per unit area was calculated as an indicator of vascular density.

### Biochemical analysis

Superoxide dismutase (SOD) and malondialdehyde (MDA) assays were performed to determine oxidative stress and extent of tissue injury, respectively. SOD and MDA assay kits were purchased from Invitrogen and Abcam, respectively. After flap extraction on day 7, 100 mg of tissue samples (1 cm × 1 cm) were collected from zone-II, washed with PBS, and processed following the manufacturer’s protocol to determine SOD and MDA contents.

### Immunohistochemistry (IHC)

Another set of tissue sections were stained with horseradish peroxidase (HRP)/3, 3‘ Diaminobenzidine (DAB) detection IHC kit (Abcam 64264, UK) following the manufacturer’s instructions. Briefly, tissue slides were boiled in sodium citrate buffer (pH 6.0) at 95 °C followed by deparaffinization and rehydration. These slides were then incubated with anti-CD34 (1:100, #ab81289, Abcam), anti-VEGF (1:100, #sc-57496, Santa Cruz), anti-CD68(1:100, #MCA341GA, Bio-Rad), and anti-CCR7 (1:100, #ab32527, Abcam) primary antibodies at 4 °C overnight after simultaneous blocking with hydrogen peroxide and protein block (10 minutes each). After applying biotinylated goat anti-polyvalent and streptavidin peroxidase, slides were incubated with DAB chromogen. Hematoxylin (H&E Stain Kit, Abcam, UK) was used for nucleus counterstaining. Slides were finally observed under a light microscope (Olympus, BX53) coupled with a camera (Olympus DP72). Percent optical density (%IOD) was measured using Fiji-ImageJ software.

### Statistical analysis

All experimental results are presented as mean ± standard deviation (SD). All statistical analyses were conducted using *t*-test of Graph Pad Prism 7. Values of *p* < 0.05 were considered statistically significant.

## Results

### Physicochemical characterization of CNH hydrogel

**Fig. 1:**
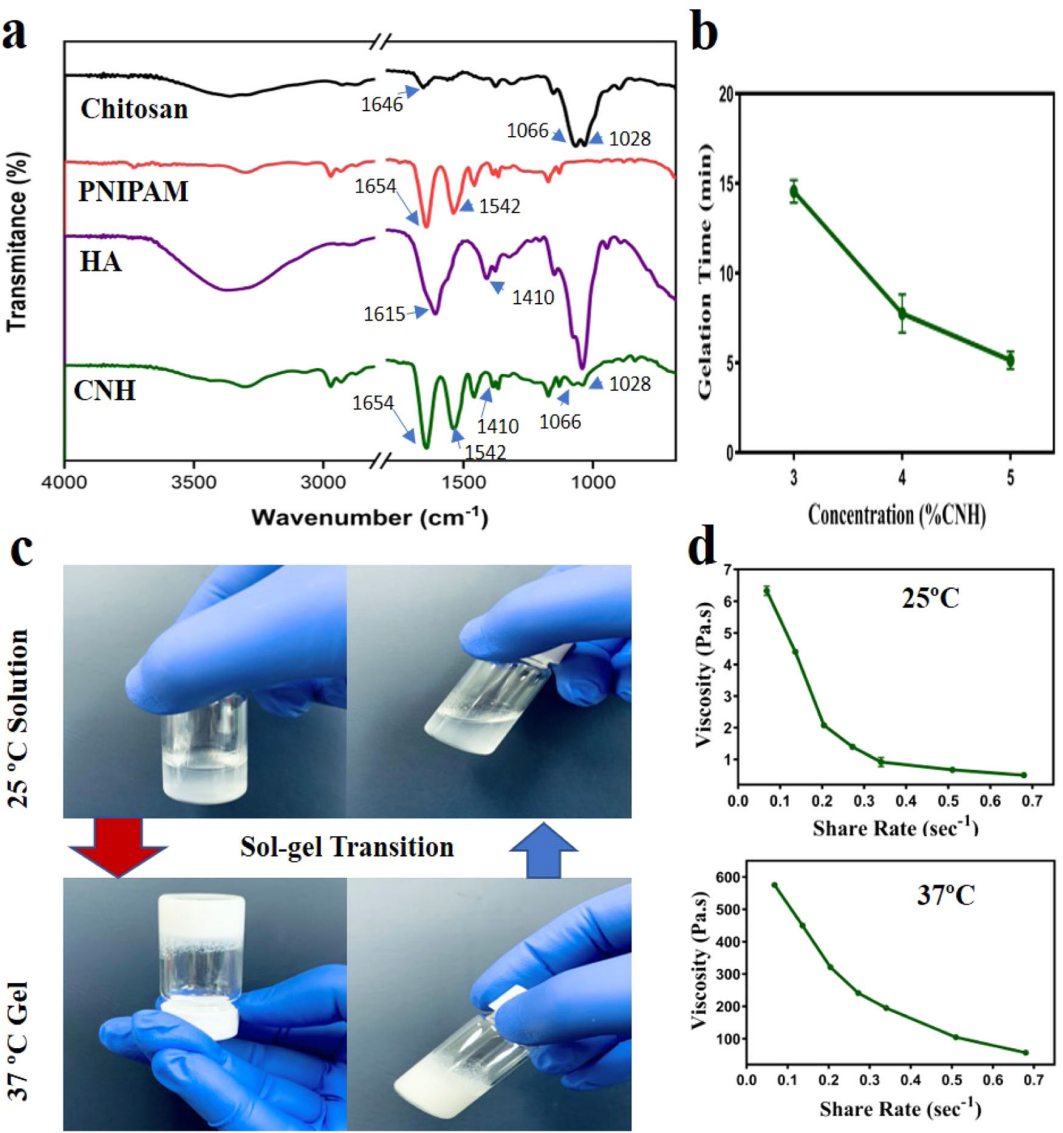
Fourier transform infrared spectroscopy (FTIR) spectra of Chitosan, PNIPAM, Hyaluronic acid and CNH co-polymer, yielded by thermal precipitation followed by EDC/NHS crosslinking with their respective absorption peaks (**a**). Concentration dependent gelation time of CNH hydrogels (**b**), Visual observation of thermosensitive behavior of 5% CNH hydrogel at 25 °C (sol-state) and 37 °C (gel-state) with their respective viscosity (**c**, **d**)

FTIR spectra of CS, PNIPAM, HA, and synthesized CNH co-polymer were analyzed to identify chemical structures. As shown in Fig. 1a, CS had peaks at 1641–1646 cm^−1^ and 1585 cm^−1^ corresponding to C = O stretching and N–H (amide II) bending vibration, respectively [22]. Additionally, it showed absorption bands at 1066 and 1028 cm^−1^ corresponding to C-O-H stretching [23, 24]. PNIPAM displayed distinctive peaks at 1654, 1542, and 1375 cm^−1^ corresponding to amide I, amide II, and a methyl group in CH(CH_3_)_2_, respectively [25]. Characteristic peaks of hyaluronic acid appeared at 1615 and 1410 cm^−1^ corresponding to asymmetric COO- stretching vibration and symmetric COO- stretching vibration, respectively. Co-polymerization of PNIPAM with chitosan and hyaluronic acid was confirmed by recognizable peaks that appeared in CNH at 1654, 1542, 1410,1066, and 1028 cm^−1^.

Figure 1b, c show sol-gel phase transition of the hydrogel as a function of temperature. By using the tube inversion method, the gelation time of different concentration of hydrogel solutions (3, 4 & 5%) was recorded where all hydrogel solutions could form opaque gel at 37 °C and flow freely inside the vial at 25 °C. Even after being held in the reverse posture for 10 seconds, hydrogels at 37 °C were entirely in gel form without falling. In this experiment, the gelation time and CNH concentration were inversely related. A higher concentration of CNH could speed up the gelation at physiological temperature (37 °C). Gelation time of 5% CNH hydrogel was found to be shorter (5.15 ± 0.49 minutes) than that of 4% CNH hydrogel (7.75 ± 1.06 minutes) or 3% CNH hydrogel (14.55 ± 0.64 minutes).

Due to the lowest observed gelation time of 5% CNH hydrogel, it was considered as the optimized sample for further study. Viscosity of this hydrogel was measured at room temperature (25 °C) and physiological temperature (37 °C). Figure 1d demonstrates viscosity of the hydrogel against shear rate. Viscosity of CNH hydrogel was 6.33 ± 0.14 Pa.s at 25 °C and 575.56 ± 1.10 Pa.s at 37 °C. Viscoelastic property of the hydrogel was confirmed by an inverse co-relation of viscosity with shear rate.

**Fig. 2:**
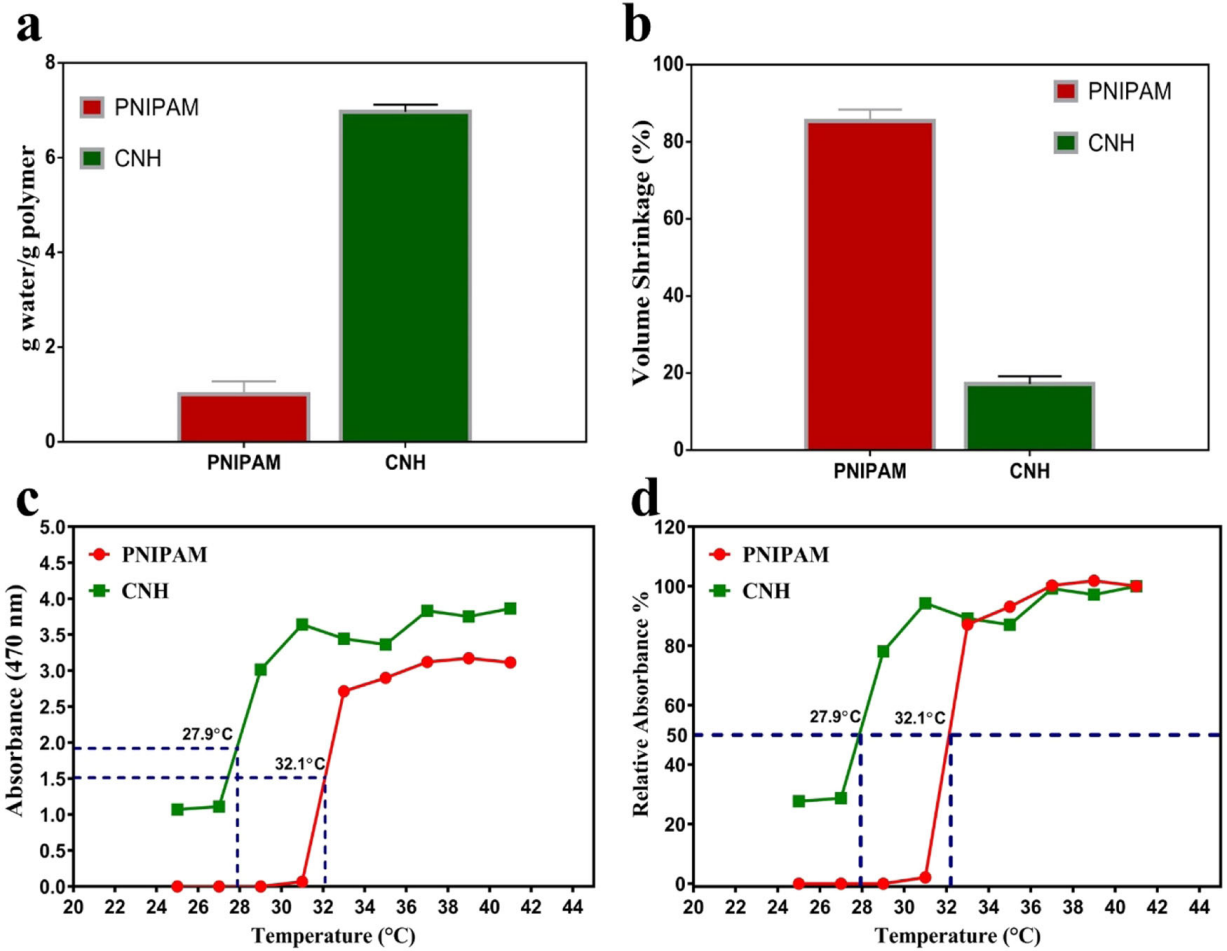
Representative histogram of water content (**a**) and volume shrinkage (**b**) of 5% (w/v) PNIPAM and 5% CNH hydrogels at 37 °C temperature. Absorbance at 470 nm (**c**) and corresponding relative absorbance (**d**) of both PNIPAM and CNH thermo-responsive hydrogels at increasing temperature

CHN is anticipated to retain more water and exhibit less volume contraction than PNIPAM since chitosan and hyaluronic acid are known to have good water absorption and retention abilities [26]. Water retention and volume shrinkage of CHN hydrogel were compared with the same concentration (5% w/v) of PNIPAM where it converted to white precipitate at 37 °C. On the other hand, CNH hydrogel gelled at the same temperature and retained higher water content. Water contents were 1.01 ± 0.27 and 6.97 ± 0.15 g water/g polymer for PNIPAM and CNH hydrogels, respectively (Fig. 2a). From 25 °C to 37 °C, volume shrinkages of PNIPAM and CNH hydrogels were 85.48 ± 3% and 17.2 ± 2%, respectively (Fig. 2b). Nearly 85% of water in the polymer solution was removed by the extreme volume shrinkage of PNIPAM, creating low water content hydrogel. On the contrary, for CNH which comprised CS and HA, the volume was changed by 17% and water content in the hydrogel was enhanced 4.9 times of PNIPAM.

By measuring the turbidity of each polymer solution with a temperature-controlled UV/VIS optical spectrophotometer, the LCST was determined from sol-gel phase transition (Fig. 2c, d). The temperature that corresponds to half of the highest change in absorbance is known as the LCST [19]. The LCST was calculated to be 27.9 °C and 32.1 °C for CNH and PNIPAM, respectively.

### In vitro cytotoxicity evaluation

**Fig. 3:**
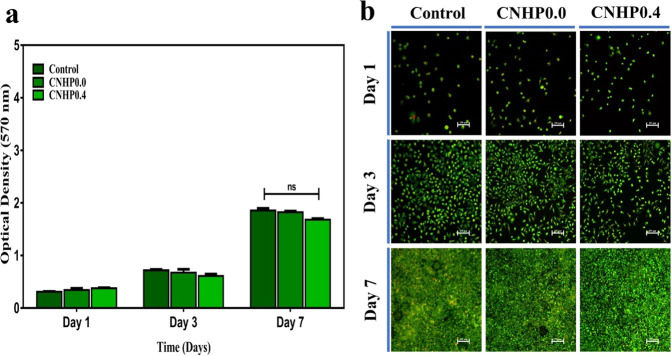
Cytotoxicity evaluation by MTT assay after 1, 3, 7 days incubation of L929 fibroblast cells with hydrogel extracted media (**a**). The cell attachment and proliferation with hydrogel extracted media after 1, 3, 7 days of incubation (**b**). F-actin and nuclei visualized by FITC and HOECHEST respectively. Scale bar 250 µm

L929 fibroblast cells were utilized to evaluate the cytocompatibility of CNHP0.4 hydrogel along with the CNHP0.0 hydrogel and control groups. Changes in optical density were measured after 1, 3, and 7 days using a colorimetric MTT assay followed by incubation with the respective extraction media. Correlated qualitative fluorescent images were assessed as shown in Fig. 3a, b. The optical density of each sample increased gradually with increasing time. However, there was a certain decrease compared to the control group due to the presence of high molecular weight HA in the composition of the hydrogel. HA can restrict fibroblast cell proliferation [27]. However, the decrease in OD value and cell proliferation rate were insignificant compared to those of the control. These results indicated non-cytotoxic characteristics of these hydrogels.

### In vitro papaverine release profile from hydrogel

**Fig. 4:**
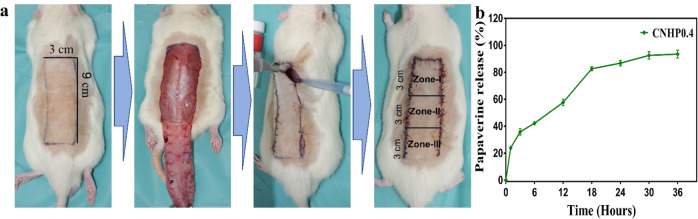
McFarlane skin flap model at rat dorsum (**a**). Complete flap elevation and immediate closure after hydrogel application on the fascia. Three equal zones were divided for post-surgical evaluation. In-vitro papaverine release profile from papaverine loaded CNHP0.4 hydrogel (**b**)

The percent papaverine release from CNHP0.4 hydrogel was determined. Results are shown in Fig. 4b. It was found that papaverine was released gradually rather than quickly. Around 90% of the drug was released during 36 hours. This release profile demonstrated the potential of controlled drug release capacity of the hydrogel.

### Effect of CNHP0.4 hydrogel on skin flap and tissue edema

**Fig. 5:**
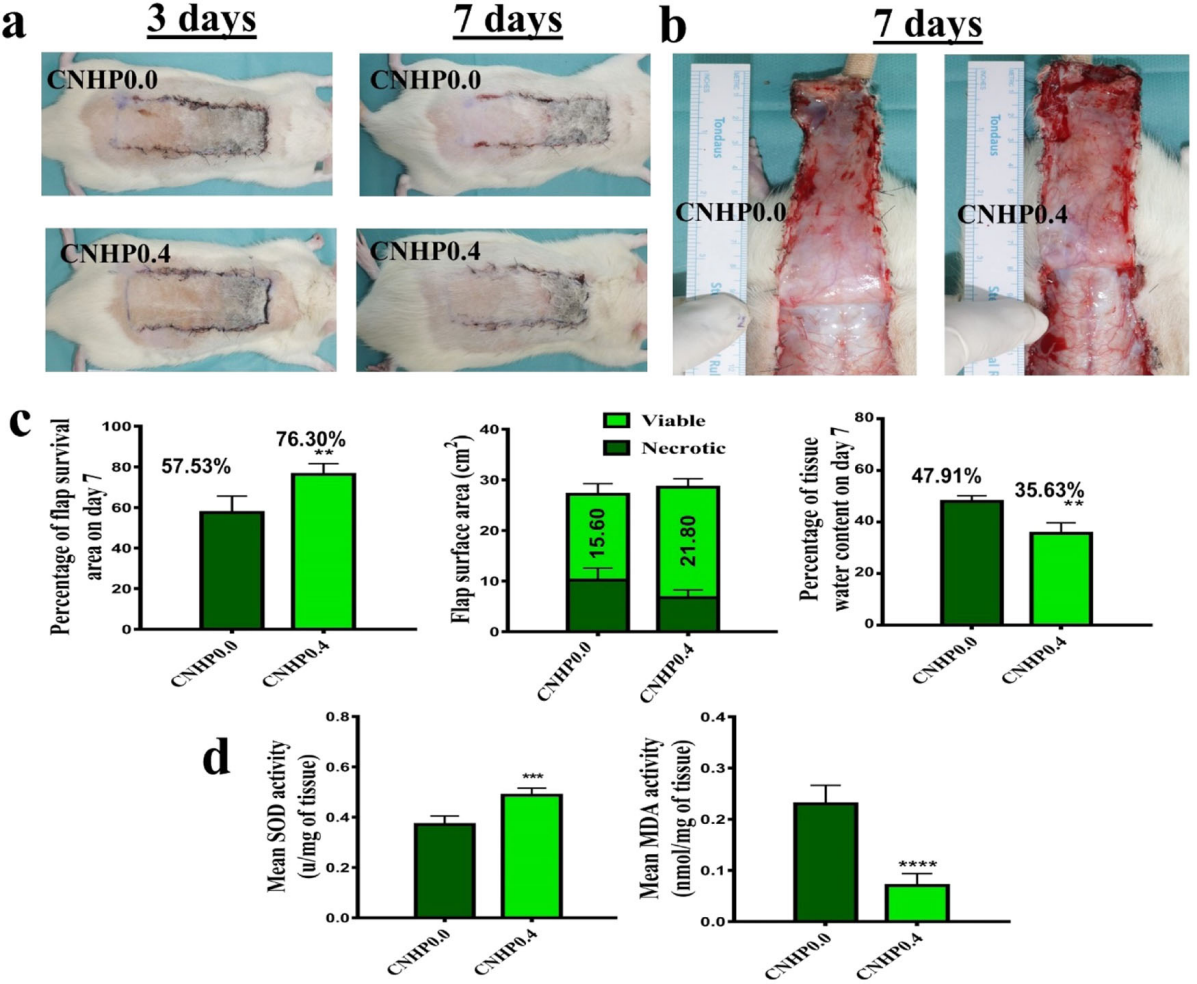
Digital photographs of skin flap survival on post operative 3 and 7 days with CNHP0.0 and CNHP0.4 hydrogel groups (**a**), Digital photographs of the inner side of skin flaps in each group on post operative day 7 showing tissue edema (**b**), Histograms showing percentage of flap survival, viable/ necrotic surface area (cm^2^) and percentage of tissue water content on post operative day 7 (**c**), Mean SOD and MDA levels in both hydrogel groups (**d**). All the values are expressed as means ± SDs (*n* = 6 per group). **p < 0.01, ***p < 0.001 and ****p < 0.0001

All flaps were observed and their color, flexibility, survival, and necrosis were noted from day 1 to day 7 following surgery. Flaps of each group were pale and bloated on the first day after surgery. On post-operative day 3, slightly dark spot started to appear at some portions of zone-I that indicated initiation of distal flap necrosis (Fig. 5a). Clearly distinguishable survival and necrotic regions were noticed on post-operative day 7. Darker necrosis spreading from zone-I to zone -II was visualized and dividing lines between survival and necrotic areas were noticed. In CNHP0.0 and CNHP0.4 hydrogels, percent flap survival areas were 57.53 ± 8.08% and 76.3 ± 5.39%, respectively, and viable flap areas were 15.6 cm^2^ and 21.8 cm^2^, respectively (Fig. 5c; *p* < 0.01). Percent tissue water content was lower in the CNHP0.4 group (35.63 ± 4.01%) than in the CNHP0.0 group (47.97 ± 2.25%; *p* < 0.01), indicating reduced tissue edema by CNHP0.4 hydrogel treatment in rat random skin flap.

### Vascularization examination in skin flap

**Fig. 6:**
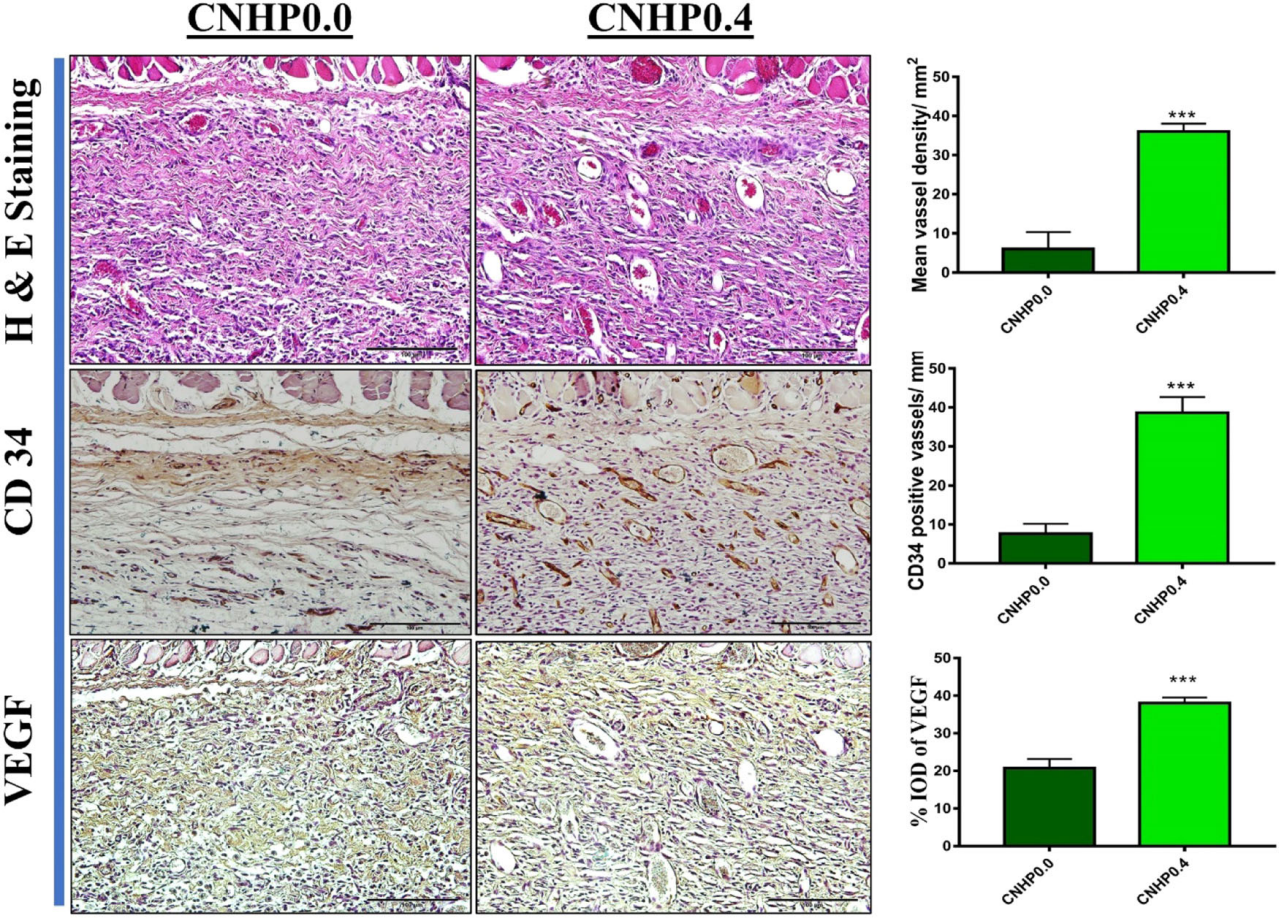
Hematoxylin and eosin (H&E) staining and immunohistochemical (IHC) staining with CD34 and VEGF of CNHP0.0 and CNHP0.4 hydrogel groups showing comparative neovascularization, CD34 positive vessels/mm and % IOD of VEGF in skin flaps along with their co-relative histogram). All the values are expressed as means ± SDs (*n* = 3 per group). ***p < 0.001

Effects of papaverine loaded CNHP0.4 hydrogel on survived skin flaps were evaluated with H&E and IHC staining to determine mean vessel density/mm^2^, CD34 positive vessel/mm^2^ and % integral optical density (IOD) of VEGF. Because of necrosis in zone-I and similar morphology of proximal flap, we analysed zone-II of each flap. CNHP0.4 hydrogel group showed better fibroblast multiplication, more newly generated granulation tissue, less inflammatory cell infiltration, and more neovascularization than control CNHP0.0 hydrogel group (Fig. 6). Mean vessel densities in CNHP0.0 and CNHP0.4 hydrogels calculated from H&E staining were 6 ± 4.35/mm^2^ and 36 ± 2.50/mm^2^, respectively. Tissue sections were stained with anti-CD34 antibody (an endothelial cell marker) that directly reflect neovascularization based on positively stained vessels/mm^2^. As showed in Fig. 6, the results of CD-34 positive vessel/mm^2^ in CNHP0.0 and CNHP0.4 were 7.66 ± 2.51/mm^2^ and 38.66 ± 4.04/mm^2^, respectively. Again, due to the ability of vessels and stromal cells to express VEGF, the expression level of VEGF was measured immunohistochemically in terms of %IOD. VEGF levels in CNHP0.0 and CNHP0.4 were 20.75 ± 2.38% and 38.02 ± 1.49%, respectively (Fig. 6; *p* < 0.001), showing increased VEGF level in the CNHP0.4 hydrogel group.

### Oxidative stress levels in skin flaps

Measurement of superoxide dismutase (SOD) activity and lipid peroxidation (MDA) content are significant biomarkers to determine oxidative stress. SOD level indicates antioxidation status and MDA content reflects the extent of tissue injury [28]. The papaverine loaded CNHP0.4 hydrogel group showed higher level of SOD (0.48 ± 0.28 u/mg of tissue) than the control hydrogel (CNHP0.0) group (0.37 ± 0.03 u/mg of tissue). On the other hand, the CNHP0.4 hydrogel group had lower MDA content (0.07 ± 0.02 nmol/mg of tissue) than the control hydrogel (CNHP0.0) group (0.20 ± 0.03 nmol/mg of tissue).

### Inflammatory cytokine levels in skin flaps

**Fig. 7:**
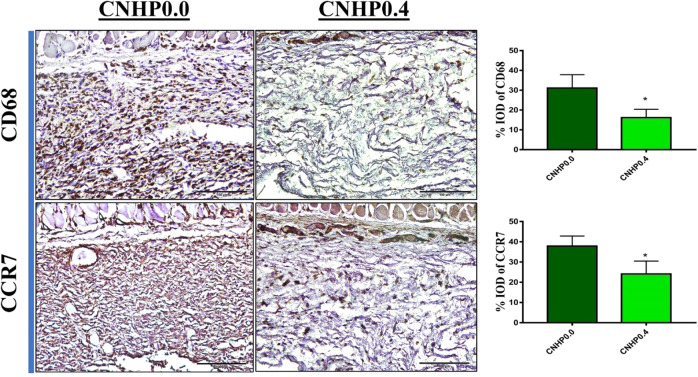
Immunohistochemical (IHC) staining of CNHP0.0 and CNHP0.4 hydrogel treated skin flaps with CD68 and CCR7 antibody showing reduced macrophage infiltration resulting suppressive inflammatory response in CNHP0.4 hydrogel treated skin flaps along with their co-relative histograms. All the values are expressed as means ± SDs (*n* = 3 per group). ***p < 0.001

Inflammatory responses in both hydrogel implanted flap groups were assessed by determining expression levels of CD68 and CCR7 known to be macrophage markers [29, 30] through immunohistochemical staining (IHC). Both markers showed significantly lower expression levels in the CNHP0.4 hydrogel group than in the CNHP0.0 group (Fig. 7). The %IOD of CD68 was 31.11 ± 6.73% in the CNHP0.0 group and 16.15 ± 4.21% in the CNHP0.4 group (*p* < 0.01). The %IOD of CCR7 was 37.90 ± 4.95% in the CNHP0.0 group and 24.11 ± 6.39% in the CNHP0.4 group (*p* < 0.01).

## Discussion

Distal end skin flap necrosis lengthens treatment procedure, increases expanse of hospitalization, and necessitates more surgical interventions in reconstructive surgery [31]. Papaverine is a well-known anti-spasmodic drug used in microsurgery as a smooth muscle relaxant and investigated for tissue expansion [32]. It has been reported that papaverine can improve micro-anastomosis and prevent blood induced vasospasm [33]. Systemic administration of papaverine is contraindicated due to the apoptotic induction ability of endothelial and smooth muscle cells [34]. Topical, intraluminal, and perivascular administration routes of drug delivery have been studied for efficiency evaluation [7]. Also, because of having short half-life, we hypothesized that loading papaverine in a hydrogel-based drug carrier system could achieve long term efficacy at the flap injury site. The aim of this study was to develop a thermosensitive hydrogel carrying papaverine for improving skin flap survival in rats. Results indicated successful co-polymerization of CS and HA to PNIPAM that made the CNH hydrogel. In vitro drug release was investigated followed by papaverine loading with the CNHP0.4 hydrogel. Significant increase in skin flap survival and decrease of tissue edema were evident at 7 days after intradermal application of the hydrogel. Furthermore, our results revealed that the CNHP0.4 hydrogel could enhance skin flap survival by increasing neo-vascularization, dampening oxidative stress and suppressing inflammation.

Controlled release of drugs can be achieved by using the sol-gel transition ability of thermo-responsive hydrogels. These stimuli-sensitive hydrogels have attracted tremendous attention in tissue regeneration studies [35]. Injectable hydrogel is capable of filling up skin wound and reduce necrosis of skin flap by administering hydrogel at solution state. In this study, CNH hydrogel was fabricated and papaverine was loaded (CNHP0.4) to observe skin flap survival. As shown in Fig. 2d, viscosity of CNH hydrogel was increased at 37 °C compared to that at 25 °C. However, shear thinning viscoelastic property was observed for both cases as viscosity was inversely proportional to shear rate. It indicated the injectability of the hydrogel at room temperature and its ability to form gel at body temperature. The LCST of PNIPAM was influenced by the addition of CS and HA into CNH hydrogel due to incorporation of hydrophilic carboxyl and hydroxyl groups [36]. Results of 50% relative absorbance indicated that the LCST of CNH was lower than that of raw PNIPAM polymer (Fig. 2d). Thus, CNH hydrogel remained in sol-state below physiological temperature and ensured induction of gel formation after in-vivo administration at flap injury site above room temperature. The gelation time was less than 5 minutes for 5% CNH hydrogel (Fig. 1b) and it was considered as optimized control hydrogel (CNHP0.0) for this study.

Hydrogels are hydrophilic in nature. They can absorb water multiple times of its dry weight at equilibrium level. Water content of a hydrogel can be a measure of transport efficiency of nutrients [9]. The CNH hydrogel contains much more water than the PNIPAM because they contain components such as CS and HA known to possess strong water absorption and water retention abilities. This may suggest that the CNH hydrogel can facilitate the adsorption and diffusion of solutes through the interior network. However, CNH successfully gelled at 37 °C and had higher water content than PNIPAM. Furthermore, PNIPAM showed higher volume shrinkage (85%) than CNH (17%) at the same temperature. Lower water content of PNIPAM was the reason behind its higher volume shrinkage caused by water discharge after gelation, an intrinsic thermo-contractive characteristic of PNIPAM. Extent of volume contraction was also maintained by the addition of CS and HA, suggesting that CS and HA were crucial in maintaining the integrity of CNH polymer hydrogel.

In order to obtain advantages from properties of both natural polymers (CS and HA), we grafted CS and HA to PNIPAM to develop a thermosensitive hydrogel (CNHP0.0) as papaverine carrier to promote vascularization in random skin flap that subsequently increased flap survival. After papaverine loading to the hydrogel (CNHP0.4), in vitro cytotoxicity evaluation was performed. The introduction of two biocompatible polymers would enhance the biocompatibility of newly grafted polymer hydrogel but there was no significant difference found in the cytotoxicity evaluation. In fact, no severe post operative complication was noticed during 7 days of study period.

Improving blood supply from proximal to distal zone of skin flaps is considered an improvement in angiogenesis which can improve skin flap survival. VEGF is an angiogenic growth factor that can promote angiopoiesis by stimulating proliferation and regeneration of vascular endothelial cells [37]. The role of VEGF in endothelial cell functions including proliferation has been identified by numerous research. VEGF can support neo-vascularization and blood flow to the ischemic skin flap, consequently increasing flap survival [38]. In the present study, VEGF expression was significantly higher and flap water content was notably lower in papaverine loaded CNHP0.4 hydrogel group than in the CNHP0.0 hydrogel group. Furthermore, mean vessel density (MVD) from H&E staining and CD34 positive vessels from IHC staining showed better neovascularization in the papaverine hydrogel (CNHP0.4) group than in the control hydrogel (CNHP0.0) group. These results suggest that papaverine can promote vascularization in rat skin flaps by VEGF induced angiogenesis, resulting in increased flap survival.

Platelet aggregation, leukocyte-endothelium interactions and oxygen-free radicals are all implicated in ischemia and necrosis when blood flow in micro vessels is compromised [39]. Oxidative stress due to increased free radical production is one of the main contributors to necrosis [40]. Superoxide dismutase (SOD), an anti-oxidant enzyme present in the body, can scavenge free radicals and prevent tissue injury. The level of SOD is a prime indicator to determine antioxidant status. Lipids of cell membrane are attacked by free radicals produced due to injury, resulting in peroxidation and destruction of cells and tissues. Malondialdehyde (MDA) is another biochemical that can reflect the extent of tissue injury which is an indicator of lipid peroxidation [41]. Consequently, SOD and MDA are two important biomarkers to determine the oxidative stress status of tissue injury. In this study, SOD level was higher and MDA content was lower in papaverine loaded CNHP0.4 hydrogel than in the control CNHP0.0 hydrogel. These findings indicated that papaverine in CNHP0.4 hydrogel increased flap survival possibly by reducing oxidative stress in rat skin flaps.

Extent of inflammatory reaction at the injury site plays a vital role in random skin flap survival. Inflammatory cell infiltration due to moderate coagulative necrosis is obvious in skin flap surgery. Greater inflammation causes pronounced necrosis, resulting in compromised success in flap survival [28]. It has been reported that papaverine is a pluripotent anti-inflammatory agent in cerebral ischemic-reperfusion injury [42] and hyper-inflammatory phase of COVID-19 patients with cardiovascular diseases [43]. In the present study, both macrophage markers (CD68 and CCR7) showed significantly decreased percentage of positive cells in flaps with papaverine loaded CNHP0.4 hydrogel than in the control CNHP0.0 hydrogel group.

Many drugs have been reported to be able to promote reversal of ischemia of skin flaps [44–46]. However, they need oral and intravascular administration daily due to their limited half-life and low plasma concentrations. On the other hand, CNHP0.4 hydrogel can be intradermally administered once in the flap during surgery. The papaverine loaded CNHP0.4 hydrogel can be transformed into gel due to its thermosensitive characteristic. Slow release of the drug in the flap injury site can reduce vasospasm and subsequently promote skin flap survival, thus increasing neo-vascularization, decreasing oxidative stressed, and decreasing inflammatory reactions.

Besides all these outcomes there are still some limitations of this study. Application in larger animal model could be done as preclinical study and could also be compared with other active molecules that have been reported previously [28, 47, 48]. These limitations could not be overcome because of limited laboratory facility. Further study is encouraged to investigate other effects of CNHP0.4 hydrogel on random skin flap survival.

## Conclusion

In conclusion, papaverine loaded thermo-responsive hydrogel CNHP0.4 has acceptable biocompatibility and applicability in random skin flaps of rats. It can increase skin flap survival in rats by promoting angiogenesis, decreasing oxidative stress, and suppressing inflammatory cytokines. All these outcomes indicate significant effectiveness of CNHP0.4 hydrogel in random skin flap surgery.

## Supplementary Information

Supplementary Information
