# Supplementary material for: Papaverine loaded injectable and thermosensitive hydrogel system for improving survival of rat dorsal skin flaps
Source: J Mater Sci Mater Med. 2023 May 20;34(6):28. doi: 10.1007/s10856-023-06732-4 (PMC10199301; doi:10.1007/s10856-023-06732-4)
Supplement: Supplementary file 1 — Supplementary Information [file 10856_2023_6732_MOESM1_ESM.docx]

**Papaverine loaded injectable and thermosensitive hydrogel system for preventing vascular spasm and improving survival of rat dorsal skin flaps**

Md Sowaib Ibne Mahbub^a^, Yeong jin Kim^c^, Byong-Taek Lee^a, b*^and Hwanjun Choi^b,c*^

*^a^ Department of Regenerative Medicine, College of Medicine, Soonchunhyang University, Cheonan, South Korea*

*^b^ Institute of Tissue Regeneration, College of Medicine, Soonchunhyang University, Cheonan, South Korea*

*^c^ Department of Plastic & Reconstructive Surgery, Soonchunhyang University,Cheonan Hospital, Cheonan, South Korea*

*Corresponding author:

Byong-Taek Lee & Hwanjun Choi

College of Medicine, Soonchunhyang University, Cheonan, South Korea,

Email: [lbt@sch.ac.kr](mailto:lbt@sch.ac.kr)/ [iprskorea@gmail.com](mailto:iprskorea@gmail.com/) Phone: +82-41-570-2427 / +82-41-570-2195

| Co-polymer name | Chitosan  (% w/v) | PNIPAM  (% w/v) | Hyaluronic Acid  (% w/v) |
| --- | --- | --- | --- |
| CNH | 0.5 | 5 | 0.2 |

**Table S1: Composition of co-polymer**

**Table S2: Composition of hydrogels**

| No | Formulation name | Chitosan  (% w/v) | PNIPAM  (% w/v) | Hyaluronic Acid (% w/v) | Freeze dried and dissolved at 5% w/v in PBS | Papavarine  (mg/ml) |
| --- | --- | --- | --- | --- | --- | --- |
| 1. | CNHP0.0 | 0.5 | 5 | 0.2 |  | 0.0 |
| 2. | CNHP0.4 | 0.5 | 5 | 0.2 |  | 0.4 |
